# Transition-Metal-Free Synthesis of Unsymmetrical Diaryl Tellurides via S_H_2 Reaction of Aryl Radicals on Tellurium

**DOI:** 10.3390/molecules27030809

**Published:** 2022-01-26

**Authors:** Yuki Yamamoto, Fumiya Sato, Qiqi Chen, Shintaro Kodama, Akihiro Nomoto, Akiya Ogawa

**Affiliations:** Department of Applied Chemistry, Graduate School of Engineering, Osaka Prefecture University, Osaka 599-8531, Japan; syb02137@edu.osakafu-u.ac.jp (Y.Y.); sugar238@outlook.jp (F.S.); scb02096@edu.osakafu-u.ac.jp (Q.C.); nomoto@chem.osakafu-u.ac.jp (A.N.)

**Keywords:** unsymmetrical diaryl telluride, transition-metal-free, arylhydrazine, S_H_2 reaction, aryl radical

## Abstract

Although diaryl tellurides are parent organotellurium compounds, their synthesis methods, especially for unsymmetrical ones, are limited. This may be due to the instability of diaryl tellurides and their synthesis intermediates under reaction conditions. Radical reactions are known to exhibit excellent functional group selectivity; therefore, we focused on a bimolecular homolytic substitution (S_H_2) reaction between the aryl radical and diaryl ditelluride. Aryl radicals are generated from arylhydrazines in air and captured by diaryl ditellurides, resulting in a selective formation of unsymmetrical diaryl tellurides with high yields. The electronic effects of the substituents on both arylhydrazines and diaryl ditellurides on the S_H_2 reaction of tellurium are also discussed in detail.

## 1. Introduction

In heteroatom chemistry, the formation of carbon–heteroatom bonds is an important fundamental reaction, and a series of transition-metal-catalyzed coupling reactions between aryl compounds and heteroatoms have been widely used for the construction of carbon–heteroatom bonds [1,2,3,4]. In recent years, however, higher purity heteroatom compounds that are used as functional molecules and pharmaceuticals have been in high demand, as there is growing concern that residual metals in the products may cause functional degradation and adverse effects on the environment and human health. Hence, the development of metal-free methods for carbon–heteroatom bond formation is strongly desired. To design such methods, we focused on bimolecular homolytic substitution (S_H_2) reactions between aryl radicals and interelement compounds bearing a heteroatom–heteroatom single bond. The concept was as follows: (1) aryl radicals were generated from arylhydrazines or triarylbismuthines using air and/or sunlight; (2) aryl radicals were smoothly captured by interelement compounds based on the characteristic features of individual heteroatom–heteroatom single bonds; and (3) the high tolerance of the radical reaction to various functional groups and solvents was advantageously utilized in developing a versatile method for forming carbon–heteroatom bonds.

**Scheme 1:**
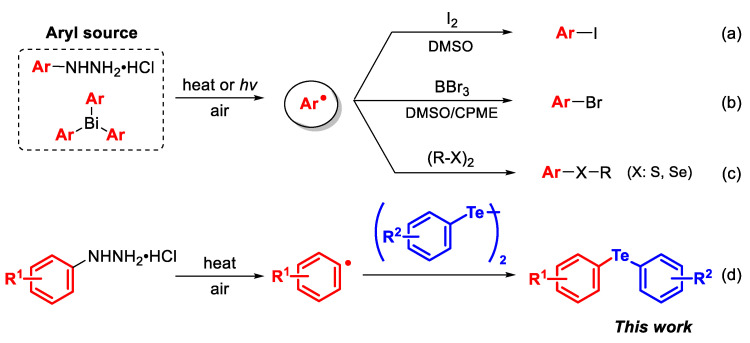
Metal-free formation of carbon–heteroatom bonds via homolytic substitution reactions between aryl radicals and interelement compounds: Synthesis of (**a**) aryl iodides, (**b**) aryl bromides, (**c**) unsymmetrical sulfides and selenides, and (**d**) unsymmetrical diaryl tellurides (this work).

We recently developed a series of carbon–heteroatom bond formation methods, as shown in Scheme 1. Aryl radicals generated in air by the oxidation of arylhydrazine hydrochlorides or photoirradiation of triarylbismuthines were successfully trapped with I_2_ or BBr_3_ in dimethyl sulfoxide (DMSO) to give the corresponding aryl halides with high yields (Scheme 1a,b) [5,6]. This system was also applicable for the formation of unsymmetrical sulfides and selenides by trapping aryl radicals with (R–X)_2_ (X = S, Se) (Scheme 1c) [7,8,9,10]. In addition, we recently reported the preliminary results regarding the trapping of aryl radicals with diaryl ditellurides (ArTe)_2_ [11]. It has been found that the S_H_2 reaction with aryl radicals (Ar’•) on tellurium can produce diaryl tellurides (Ar’–Te–Ar). Interestingly, the formed Ar’–Te–Ar can further react with another aryl radical, Ar”•, under certain conditions to yield unsymmetrical diaryl tellurides (Ar’–Te–Ar”) (Scheme 1d). Such a radical substitution reaction was not observed in sulfide or selenide synthesis, and this reaction was considered to be characteristic of tellurium [12,13], which has a low binding energy for the carbon–tellurium bond. However, the scope and synthetic utility of this S_H_2 reaction between diaryl ditellurides and aryl radicals have not yet been elucidated. 

Hence, in this study, we report the scope of the substrate application of the S_H_2 reaction between aryl radicals and diaryl ditellurides in detail.

## 2. Results and Discussion

Diaryl tellurides have attracted the attention of many researchers because of their utilization in synthetic intermediates (e.g., tellurium-mediated living radical polymerization [14,15] and catalysts for photooxidation [16,17,18]). Furthermore, their fluorescence properties and synthetic application have been actively studied [19,20,21,22,23,24,25]. Although diaryl tellurides are parent organotellurium compounds [26], their methods of synthesis, especially for unsymmetrical ones, are limited. For the synthesis of diaryl tellurides, transition-metal-catalyzed methods with diaryl ditellurides have been reported in recent decades [27,28,29,30,31,32,33,34,35,36,37,38,39]. Most of them must be carried out in an inert gas atmosphere using the Schlenk technique. In addition, to use the products as functional materials, it was necessary to remove the metals. Therefore, the development of a metal-free, simple, and efficient method for the selective synthesis of unsymmetrical and easily manipulated diaryl tellurides was strongly needed.

Since radical reactions are known to exhibit excellent functional group tolerance, the S_H_2 reaction between aryl radicals and diaryl ditellurides will provide a wide range of unsymmetrical and symmetrical diaryl tellurides with high product selectivity. In our system, aryl radicals are generated from arylhydrazines in air and captured by diaryl ditellurides, and the procedure is simple, without the use of any transition-metal reagents. Thus, clarifying the electronic effects of the substituents on both arylhydrazines and diaryl ditellurides on the S_H_2 reaction on tellurium will lead to the facile synthesis of unsymmetrical diaryl tellurides, making it a versatile alternative to conventional methods.

Considering our previous study on the S_H_2 reaction of aryl radicals on tellurium [11], optimization of the reaction conditions for the synthesis of unsymmetrical tellurides was conducted using 4-nitrophenylhydrazine hydrochloride **1a** and diphenyl ditelluride **2a** as the model substrates and Et_3_N as the base for the deprotonation of **1a**, respectively (Table 1). When the reaction was conducted in MeOH, the corresponding unsymmetrical telluride, namely, 4-nitrophenyl phenyl telluride **3a**, was obtained in a 52% yield (Entry 1). Extending the reaction time to 48 h did not improve the yield of **3a** (Entry 2). The optimization of the solvent revealed that benzene was the most effective (Entries 3–8). Increasing the loading of **1a** gradually improved the yield of **3a**, and the use of 1.0 mmol of **1a** resulted in a 74% yield of **3a** (Entries 9–10). The concentration of the substrates in benzene was also investigated in detail, and 3.0 mL of the solvent yielded the best results (Entry 8 vs. Entries 11–12). When 2,2,6,6-tetramethylpiperidine-*N*-oxyl (TEMPO) was added as a radical scavenger, the yield of **3a** dramatically decreased compared with the optimal condition, which strongly indicated that the reaction proceeded through a free-radical pathway (Entry 10 vs. Entry 13).

Based on the optimal conditions (Entry 10 in Table 1), the reaction scope for the synthesis of unsymmetrical diaryl tellurides was examined using arylhydrazine hydrochlorides and diaryl ditellurides; the results are summarized in Table 2. Arylhydrazine hydrochlorides with *p*-nitro, *p*-cyano, and *p*-trifluoromethyl groups were successfully transformed into the corresponding unsymmetrical diaryl tellurides in moderate-to-good yields using (PhTe)_2_ as the substrate (**3a**–**3c**). *o*-Nitro, *o*-methoxy, and *m*-methoxyphenylhydrazine hydrochlorides were tolerated under the reaction conditions, resulting in the corresponding diaryl tellurides in 42–58% yields, respectively (**3d**–**3f**). When *o*-methoxyphenylhydrazine hydrochloride **1e** was used as the substrate, bis(2-methoxyphenyl) telluride (**3k**, see Scheme 3a) was obtained in a 9% yield as the byproduct, owing to the S_H_2 reaction between the *o*-methoxyphenyl radical (from **1e**) and diaryl telluride **3e** (the byproduct **3k** could easily be removed from the desired unsymmetrical telluride **3e** by preparative TLC). The present unsymmetrical diaryl telluride synthesis could be applied to the synthesis of symmetrical diaryl tellurides, and **3g** and **3h** were thus obtained with high yields. Concerning the substituents of diaryl ditellurides **2**, either electron-donating groups or halogen groups at the *para*-positions of the aromatic rings were tolerated, and the corresponding unsymmetrical tellurides were obtained in high yields, using **1a** as the aryl radical source (**3i** and **3j**). As shown in Table 2, this diaryl telluride synthesis method can be applied to a wide range of arylhydrazine hydrochlorides **1** and diaryl ditellurides **2**; therefore, it enables the practical synthesis of unsymmetrical and symmetrical diaryl tellurides **3**. Considering the limited examples of unsymmetrical synthesis methods [30,40,41,42], it is noteworthy that unsymmetrical tellurides **3** were selectively obtained by the present S_H_2 reaction.

**Scheme 2:**
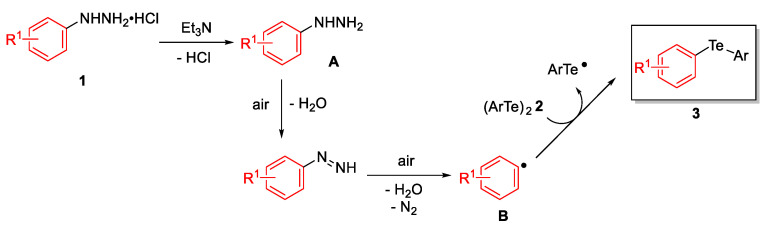
Proposed reaction pathways for the formation of unsymmetrical tellurides **3**.

Based on the control experiment (Entry 13 in Table 1) and our previous studies, possible reaction pathways for the formation of unsymmetrical diaryl telluride **3** from arylhydrazine hydrochlorides and diaryl ditellurides were proposed. Initially, the deprotonation of **1** occurred with Et_3_N to form free arylhydrazines **A** that were oxidized in air to form aryl radicals **B**. The generated aryl radicals **B** were trapped with diaryl ditellurides, which have stronger carbon-radical-trapping abilities than disulfides and diselenides, and the corresponding unsymmetrical diaryl tellurides **3** were formed via bimolecular homolytic substitution on tellurium [43,44,45,46,47] (Scheme 2).

**Scheme 3:**
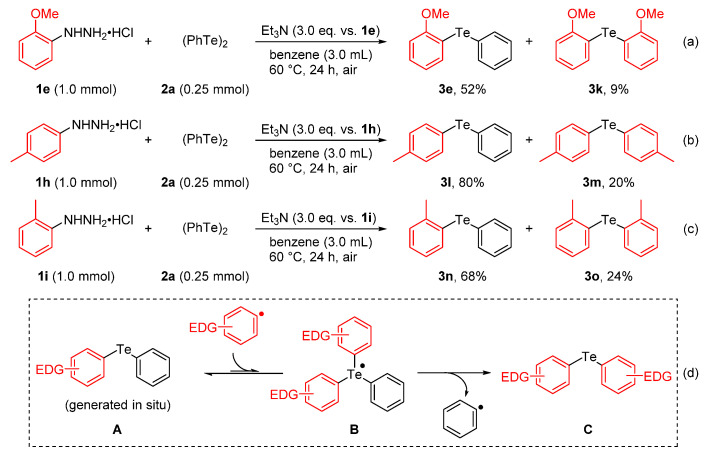
Relative reactivities of arylhydrazine hydrochlorides with electron-donating groups (EDG; (**a**) *o*-methoxy, (**b**) *p*-methyl, and (**c**) *o*-methyl) toward in situ generated (**d**) (PhTe)_2_ and diaryl tellurides.

Interestingly, in the S_H_2 reaction using arylhydrazine hydrochloride with an electron-donating group and diphenyl telluride, unsymmetrical telluride (cf. synthesis of unsymmetrical tellurides by ionic mechanism: [48,49]) is also formed in preference to symmetrical telluride (Scheme 3). Focusing on the difference in the electron-donating properties of each substituent, the yield of the byproduct, symmetrical telluride, was lower for the methoxy group than for the methyl group, and the unsymmetrical telluride was selectively obtained when **1e** was used as the aryl source (Scheme 3a vs. Scheme 3b,c). This difference could be explained by the stability of the radical species generated. As shown in Scheme 3d, unsymmetrical diaryl telluride **A** (generated in situ from arylhydrazine and diaryl ditelluride) might react with another aryl radical to form **B**. Radical species **B** is known to be destabilized by an electron-donating group, resulting in a shift of equilibrium between **A** and **B** to the left. When 2-methoxyphenylhydrazine hydrochloride **1e**, which has a stronger electron-donating property than the methyl group, is used, the formation of symmetrical telluride along with the removal of phenyl radical from **B** is expected to proceed much slower than the case of using **1h** or **1i** as the aryl radical sources.

## 3. Materials and Methods

### 3.1. General Information

Unless otherwise stated, all starting materials were purchased from commercial sources and used without further purification. Ditellurides **2b** and **2c** were synthesized by the previously reported method [50]. All solvents were distilled before use. ^1^H NMR spectra were recorded in CDCl_3_ using the JEOL JNM-ECX400 (400 MHz) FT NMR, JEOL JNM-ECS400 (400 MHz) FT NMR (Tokyo, Japan), and the Bruker BioSpin Ascend 400 spectrometer (400 MHz) (Tokyo, Japan) with Me_4_Si as the internal standard. ^13^C{^1^H} NMR spectra were recorded in CDCl_3_ using the JEOL JNM-ECX400 (100 MHz) FT NMR, JEOL JNM-ECS400 (100 MHz) FT NMR, and the Bruker BioSpin Ascend 400 spectrometer (100 MHz). The characterization data of compounds are shown as follows (^1^H and ^13^C{^1^H} NMR spectra are included in the Supplementary Materials).

### 3.2. General Procedure for Synthesis of Diaryl Tellurides from Arylhydrazine Hydrochlorides and Diaryl Ditellurides under Air

Arylhydrazine hydrochloride **1** (1.0 mmol), diaryl ditellurides **2** (0.25 mmol), Et_3_N (3.0 eq. vs. **1**), and benzene (3.0 mL) were added to a 30 mL flask equipped with a stir bar. The resulting solution was stirred at 60 °C in open air for 24 h. After the reaction was complete, the solvent was removed under reduced pressure. The residue was dissolved in AcOMe (10 mL) and filtered using silica gel. The filtrate was concentrated under reduced pressure, and the resulting residue was purified by preparative thin-layer chromatography (eluent: CH_2_Cl_2_/*iso*-hexane) to afford the corresponding unsymmetrical or symmetrical diaryl tellurides **3**.

*(4-Nitrophenyl)(phenyl)tellane* (**3a**) (CAS: 79424-71-8) [35]. Yellow oil, 107.4 mg, 66%; ^1^H NMR (400 MHz, CDCl_3_): δ 7.94 (d, *J* = 8.2 Hz, 2H), 7.85 (d, *J* = 7.2 Hz, 2H), 7.55 (d, *J* = 8.6 Hz, 2H), 7.45–7.41 (m, 1H), 7.32 (t, *J* = 7.7 Hz, 2H); ^13^C{^1^H} NMR (100 MHz, CDCl_3_): δ 147.2, 140.4, 135.3, 130.3, 129.5, 128.2, 123.8, 112.8.

*4-(Phenyltellanyl)benzonitrile* (**3b**) (CAS: 105851-02-3) [33]. Yellow oil, 63.9 mg, 42%; ^1^H NMR (400 MHz, CDCl_3_): δ 7.85–7.82 (m, 2H), 7.57–7.54 (m, 2H), 7.44–7.37 (m, 3H), 7.34–7.29 (m, 2H); ^13^C{^1^H} NMR (100 MHz, CDCl_3_): δ 140.2, 135.8, 132.3, 130.2, 129.3, 124.5, 118.8, 112.9, 110.7.

*(2-Nitrophenyl)(phenyl)tellane* (**3d**) (CAS: 79424-69-4) [31]. Red oil, 66.7 mg, 41%; ^1^H NMR (400 MHz, CDCl_3_): δ 8.41 (d, *J* = 7.8 Hz, 1H), 7.97 (dd, *J* = 7.9, 1.1 Hz, 2H), 7.54–7.50 (m, 1H), 7.41 (t, *J* = 7.4 Hz, 2H), 7.34–7.30 (m, 1H), 7.27–7.21 (m, 2H); ^13^C{^1^H} NMR (100 MHz, CDCl_3_): δ 147.7, 141.4, 134.4, 133.5, 130.2, 129.7, 126.8, 126.3, 123.0, 118.2.

*(2-Methoxyphenyl)(phenyl)tellane* (**3e**) (CAS: 105152-01-0) [36]. Yellow oil, 62.4 mg, 40%; ^1^H NMR (400 MHz, CDCl_3_): δ 7.90 (d, *J* = 6.9 Hz, 2H), 7.43–7.38 (m, 1H), 7.30 (t, *J* = 7.6 Hz, 2H), 7.20–7.15 (m, 1H), 6.94 (dd, *J* = 7.6, 1.6 Hz, 1H), 6.81–6.79 (m, 1H), 6.76–6.72 (m, 1H), 3.88 (s, 3H); ^13^C{^1^H} NMR (100 MHz, CDCl_3_): δ 158.2, 141.3, 133.6, 129.7, 128.7, 128.2, 122.5, 112.1, 109.7, 107.8, 56.0.

*(3-Methoxyphenyl)(phenyl)tellane* (**3f**) (CAS: 1200113-82-1) [39]. Brown oil, 58.5 mg, 36%; ^1^H NMR (400 MHz, CDCl_3_): δ 7.73–7.70 (m, 2H), 7.32–7.20 (m, 5H), 7.12 (t, *J* = 8.0 Hz, 1H), 6.83–6.80 (m, 1H), 3.75 (s, 3H); ^13^C{^1^H} NMR (100 MHz, CDCl_3_) δ 160.0, 138.3, 130.3, 130.2, 129.6, 128.0, 123.1, 115.5, 114.6, 113.9, 55.3.

*Diphenyltellane* (**3g**) (CAS: 1202-36-4) [11]. Yellow oil, 122.6 mg, 87%; ^1^H NMR (400 MHz, CDCl_3_): δ 7.69 (m, 4H), 7.26 (m, 2H), 7.19 (m, 4H); ^13^C{^1^H} NMR (100 MHz, CDCl_3_): δ 138.0, 129.6, 127.9, 114.7.

*Di-p-tolyltellane* (**3h**) (CAS: 834-15-1) [42]. Beige solid, 115.8 mg, 75%; ^1^H NMR (400 MHz, CDCl_3_): δ 7.57 (d, *J* = 8.1 Hz, 4H), 7.01 (d, *J* = 7.6 Hz, 4H), 2.31 (s, 6H); ^13^C{^1^H} NMR (100 MHz, CDCl_3_): δ 138.1, 137.8, 130.4, 110.8, 21.3.

*(4-Nitrophenyl)(p-tolyl)tellane* (**3i**). Yellow solid, mp 65.0–66.0 °C, 127.3 mg, 75%; ^1^H NMR (400 MHz, CDCl_3_): δ 7.94 (d, *J* = 8.8 Hz, 2H), 7.76 (d, *J* = 8.0 Hz, 2H), 7.53 (d, *J* = 8.8 Hz, 2H), 7.15 (d, *J* = 7.7 Hz, 2H), 2.40 (s, 3H); ^13^C{^1^H} NMR (100 MHz, CDCl_3_): δ 147.1, 140.7, 139.8, 134.8, 131.1, 128.6, 123.7, 108.6, 21.4; HRMS (EI) m/z calcd for C_13_H_11_NO_2_Te [M]^+^: 342.9852, found: 342.9844.

*(4-Chlorophenyl)(4-nitrophenyl)tellane* (**3j**) (CAS: 2119610-21-6) [35]. Yellow solid, 127.7 mg, 71%; ^1^H NMR (400 MHz, CDCl_3_): δ 7.98 (m, 2H), 7.77 (m, 2H), 7.59 (m, 2H), 7.29 (m, 2H); ^13^C{^1^H} NMR (100 MHz, CDCl_3_): δ 147.4, 141.6, 136.1, 135.5, 130.5, 127.1, 123.9, 110.2.

## 4. Conclusions

We reported a novel transition-metal-free synthesis of unsymmetrical diaryl tellurides via the S_H_2 reaction of aryl radicals (generated from arylhydrazine hydrochlorides with bases in air) on tellurium (i.e., diaryl ditellurides or diaryl tellurides). This system could be applied to a broad substrate scope of arylhydrazines and ditellurides to produce the corresponding unsymmetrical and symmetrical diaryl tellurides in moderate to excellent yields. The electronic effects of the substituents on both arylhydrazines and diaryl ditellurides on the S_H_2 reaction on tellurium were also discussed in detail, which led to a novel strategy for the synthesis of unsymmetrical diaryl tellurides based on the difference in the reactivity of aryl radicals (from arylhydrazines) in the S_H_2 reaction on tellurium. This study has revealed a new aspect of heteroatom chemistry toward the reactivities of aryl radicals, and provides a new synthetic strategy for carbon–heteroatom bond formation for utilization in pharmaceutical and material sciences.

## Figures and Tables

**Table 1:**
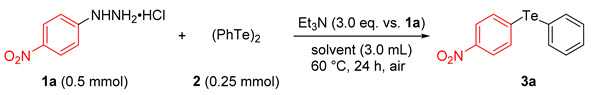
The optimization of reaction conditions for the synthesis of unsymmetrical tellurides **3a**.

Entry	Solvent	Yield of 3a (%) ^a^
1	MeOH	52
2 ^b^	MeOH	54
3	*^i^*PrOH	58
4	CH_3_CN	57
5	AcOEt	61
6	1,4-Dioxane	54
7	Toluene	57
8	Benzene	65
9 ^c^	Benzene	72
10 ^d^	Benzene	74 (66)
11 ^e^	Benzene	67
12 ^f^	Benzene	56
13 ^d,g^	Benzene	27

^a^ Determined by ^1^H NMR spectroscopy based on **2** using 1,3,5-trioxane as an internal standard (isolated yield indicated in parentheses); ^b^ reaction time: 48 h; ^c^ **1a** (0.75 mmol) was used; ^d^ **1a** (1.0 mmol) was used; ^e^ benzene (1.5 mL) was used; ^f^ benzene (6.0 mL) was used; ^g^ TEMPO (5 eq. vs. **1a**) was added.

**Table 2:**
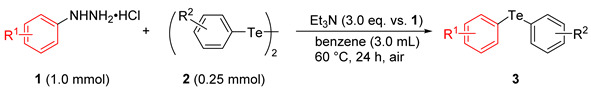
Substrate scope for the synthesis of unsymmetrical tellurides **3** from arylhydrazine hydrochlorides **1** and diaryl ditellurides **2** under air.

Entry	Arylhydrazine•HCl 1	Ditelluride 2	Product 3		Yield (%) ^a^
1	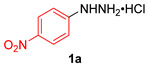	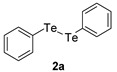	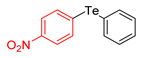	**3a**	74 (66)
2	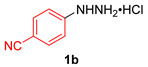	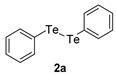	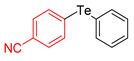	**3b**	(42)
3	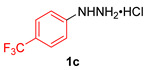	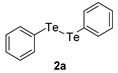	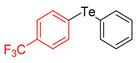	**3c**	37
4	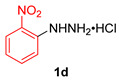	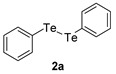	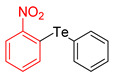	**3d**	52 (41)
5	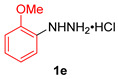	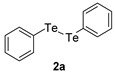	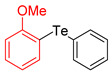	**3e**	52 ^b^ (40)
6	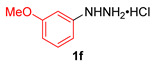	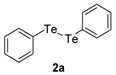	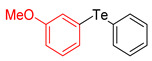	**3f**	42 (36)
7	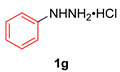	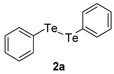	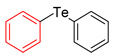	**3g**	97 (87)
8	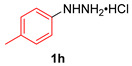	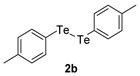	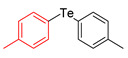	**3h**	100 (75)
9	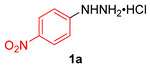	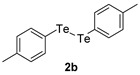	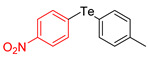	**3i**	82 (75)
10	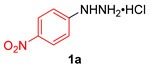	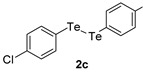	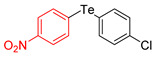	**3j**	77 (71)

^a^ Determined by ^1^H NMR spectroscopy based on **2** using 1,3,5-trioxane as an internal standard (isolated yield indicated in parentheses). ^b^ Bis(2-methoxyphenyl) telluride was obtained in a 9% yield as the byproduct.

## Data Availability

The data presented in this study are available in the article and in its Supplementary Materials.

## Supplementary Materials

The following are available online, Copies of ^1^H and ^13^C{^1^H} NMR spectra.

## Sample Availability

Samples of the compounds are not available from the authors.
