# Supplementary material for: Transition-Metal-Free Synthesis of Unsymmetrical Diaryl Tellurides via SH2 Reaction of Aryl Radicals on Tellurium
Source: Molecules. 2022 Jan 26;27(3):809. doi: 10.3390/molecules27030809 (PMC8839872; doi:10.3390/molecules27030809)
Supplement: Supplementary file 1 [file molecules-27-00809-s001.zip › molecules-1549170-supplementary.pdf]

molecules

Supplementary Materials

Transition-Metal-Free Synthesis of Unsymmetrical Diaryl Tellurides via S_H2 Reaction of Aryl Radicals on Tellurium

Yuki Yamamoto, Fumiya Sato, Qiqi Chen, Shintaro Kodama *, Akihiro Nomoto and Akiya Ogawa *

Department of Applied Chemistry, Graduate School of Engineering, Osaka Prefecture University, Osaka 599-8531, Japan, syb02137@edu.osakafu-u.ac.jp (Y.Y.); szb02060@edu.osakafu-u.ac.jp (F.S.); scb02096@edu.osakafu-u.ac.jp (Q.C.); nomoto@chem.osakafu-u.ac.jp (A.N.)

* Correspondence: skodama@chem.osakafu-u.ac.jp (S.K.); ogawa@chem.osakafu-u.ac.jp (A.O.)

Contents

Copies of ^{1}H and ^{13}C{^{1}H} NMR spectra ···2–10

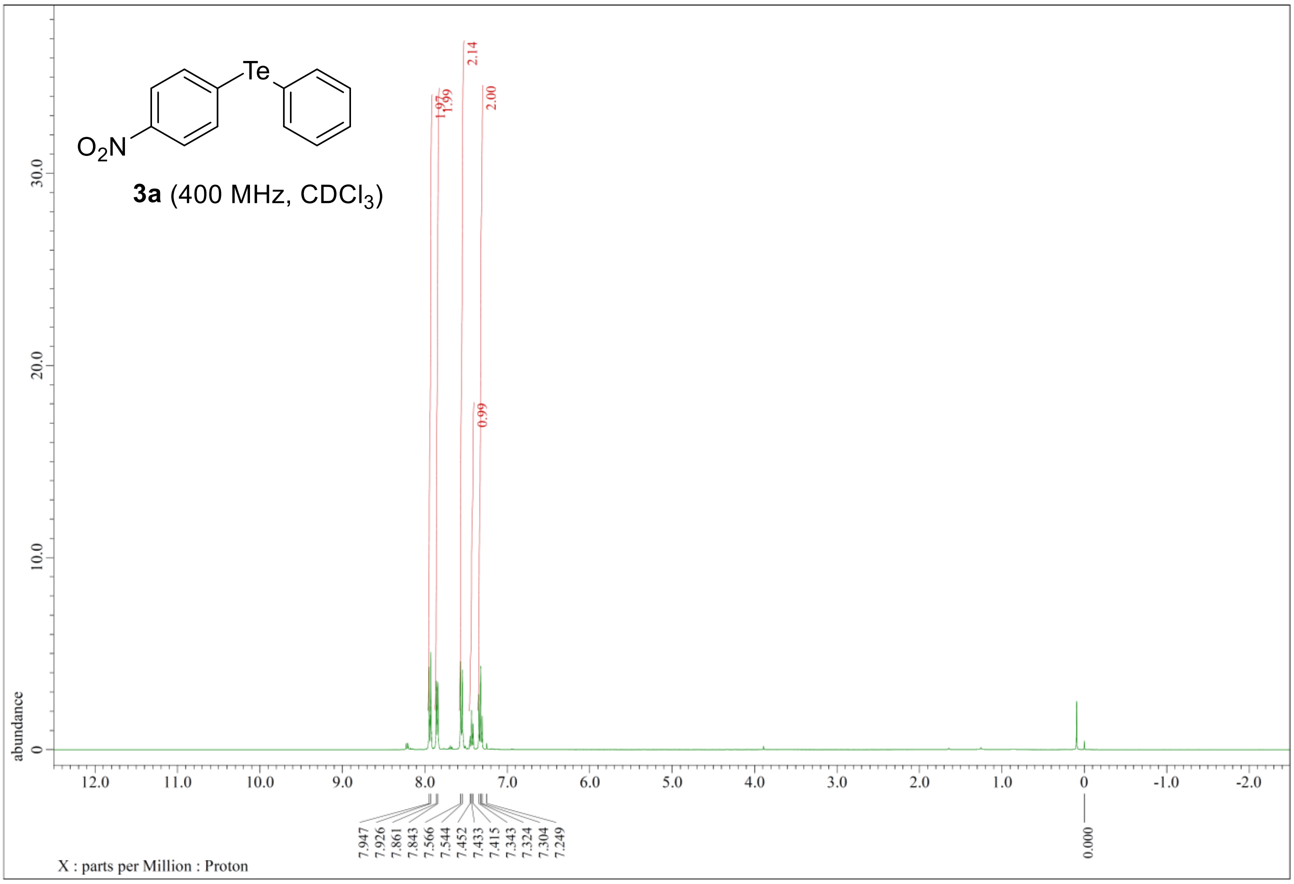
Te
O2N
3a (400 MHz, CDCl3)
abundance
X : parts per Million : Proton
7.947
7.926
7.861
7.843
7.566
7.544
7.452
7.433
7.415
7.343
7.324
7.304
7.249
0.000

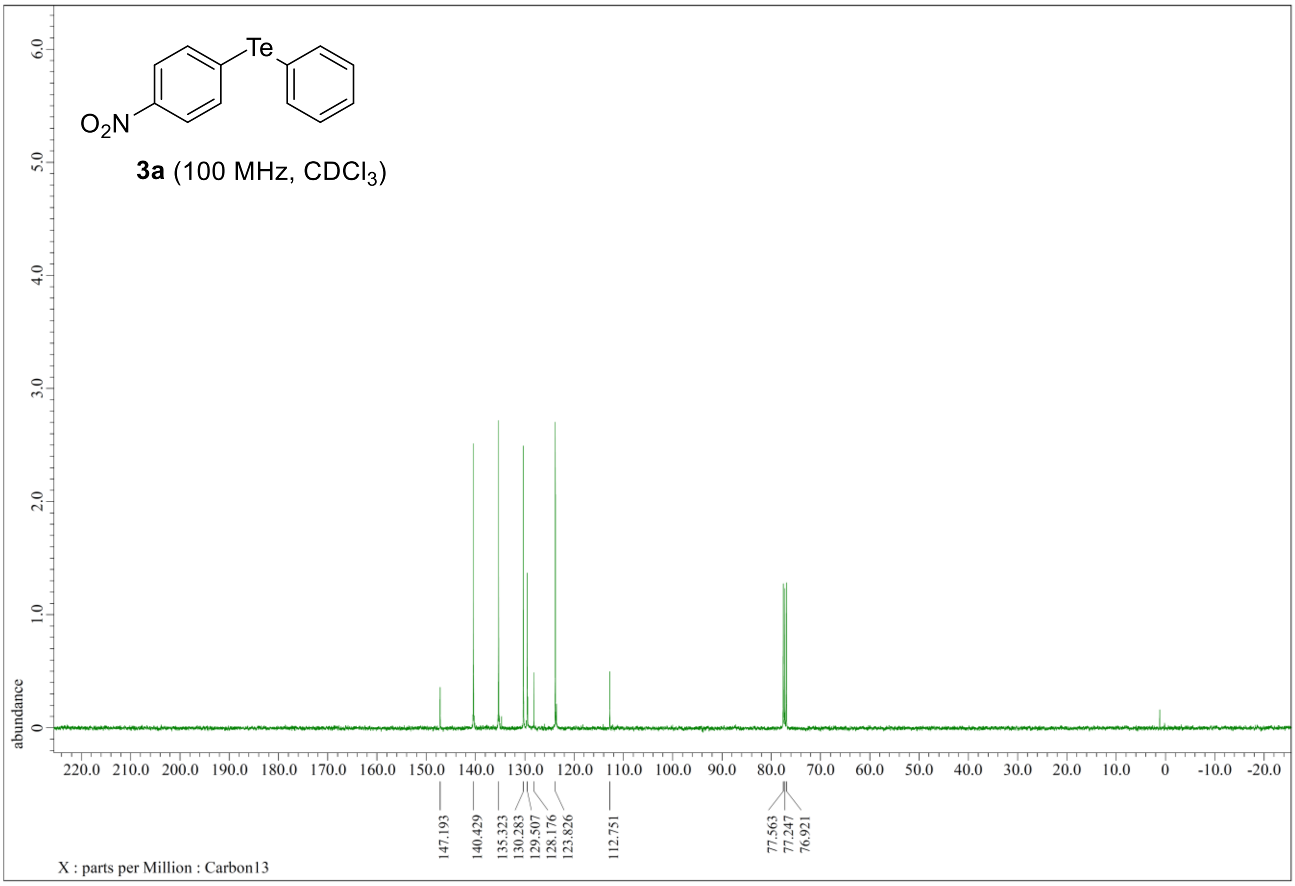
Te
O2N
3a (100 MHz, CDCl3)
abundance
X : parts per Million : Carbon13
147.193
140.429
135.323
130.283
129.507
128.176
123.826
112.751
77.563
77.247
76.921

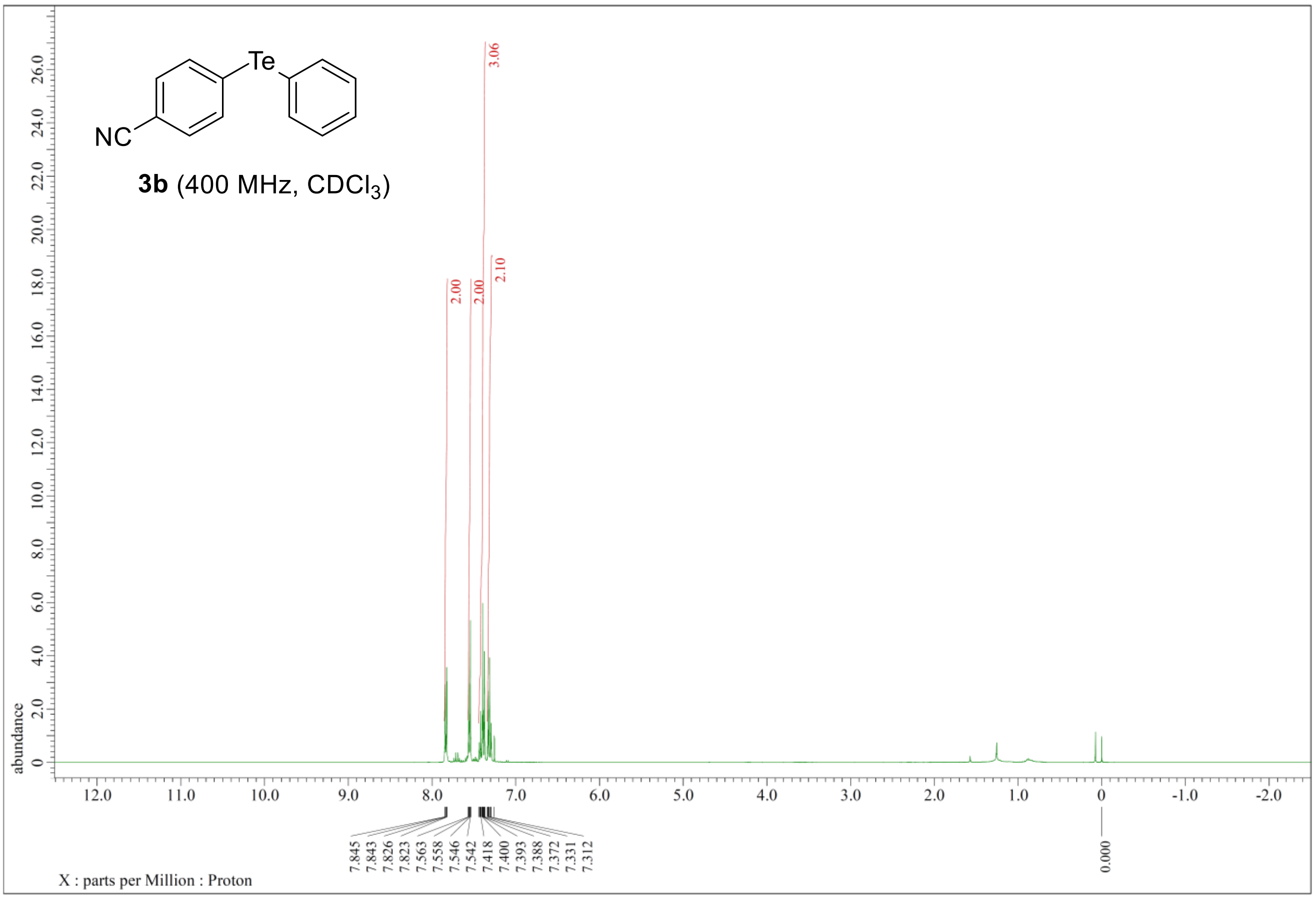
Te
NC
3b (400 MHz, CDCl3)
abundance
X : parts per Million : Proton
3.06
2.00
2.00
2.10
7.845
7.843
7.826
7.823
7.563
7.558
7.546
7.542
7.418
7.400
7.393
7.388
7.372
7.331
7.312
0.000

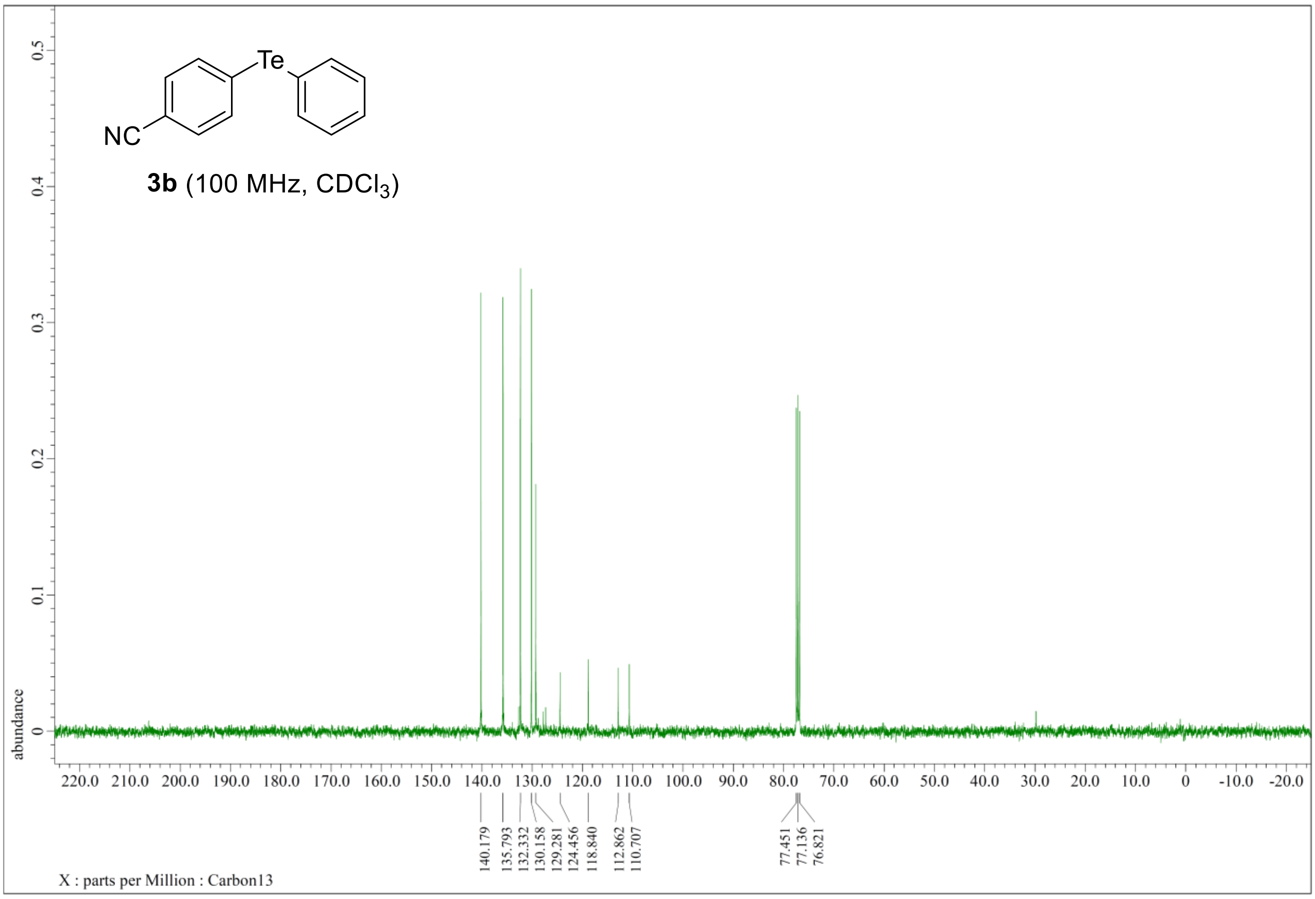
Te
NC
3b (100 MHz, CDCl3)
abundance
X : parts per Million : Carbon13
140.179
135.793
132.332
130.158
129.281
124.456
118.840
112.862
110.707
77.451
77.136
76.821

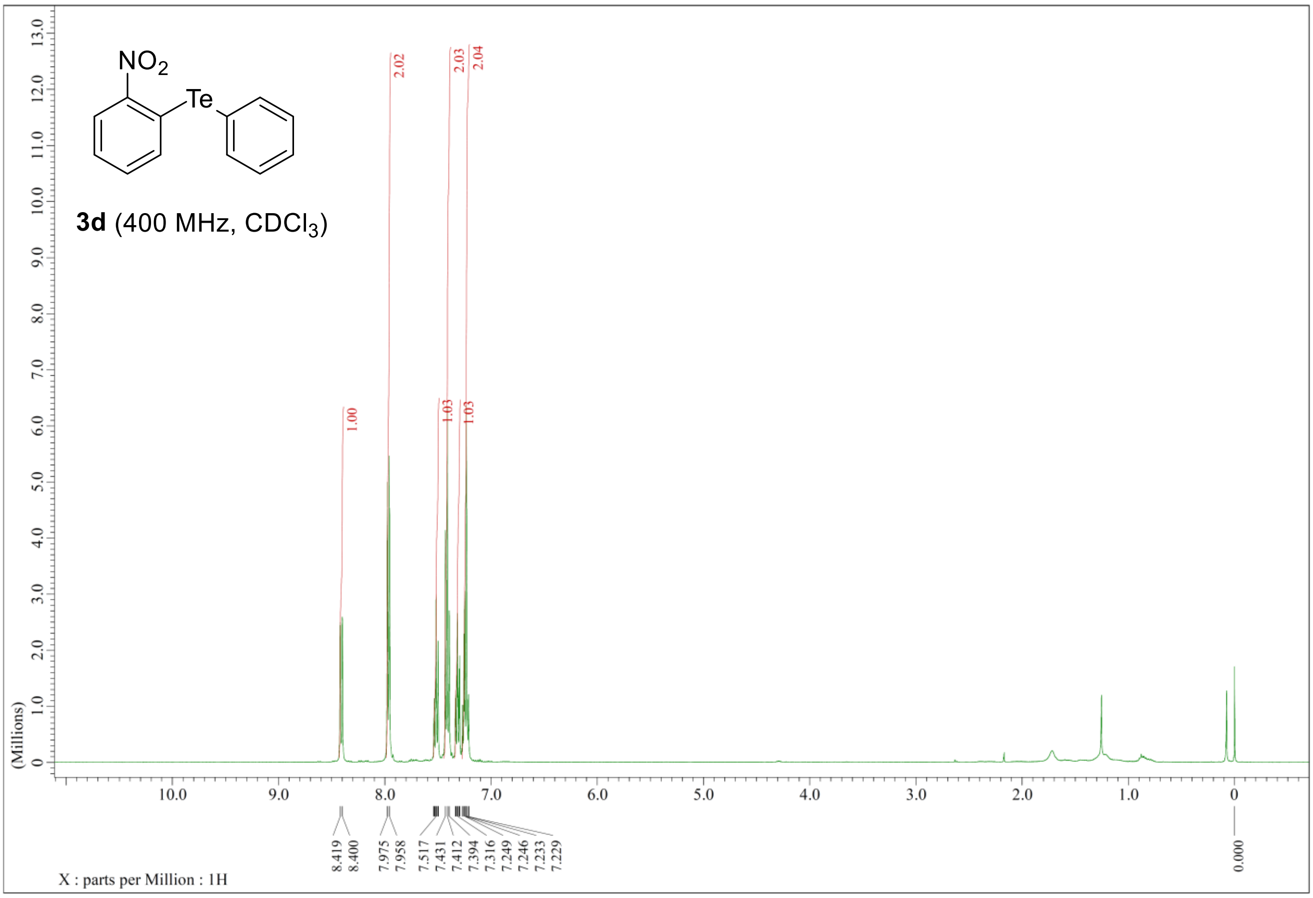
NO2
Te
3d (400 MHz, CDCl3)
1.00
2.02
1.03
2.03
1.03
2.04
8.419
8.400
7.975
7.958
7.517
7.431
7.412
7.394
7.316
7.249
7.246
7.233
7.229
0.000
(Millions)
X : parts per Million : 1H

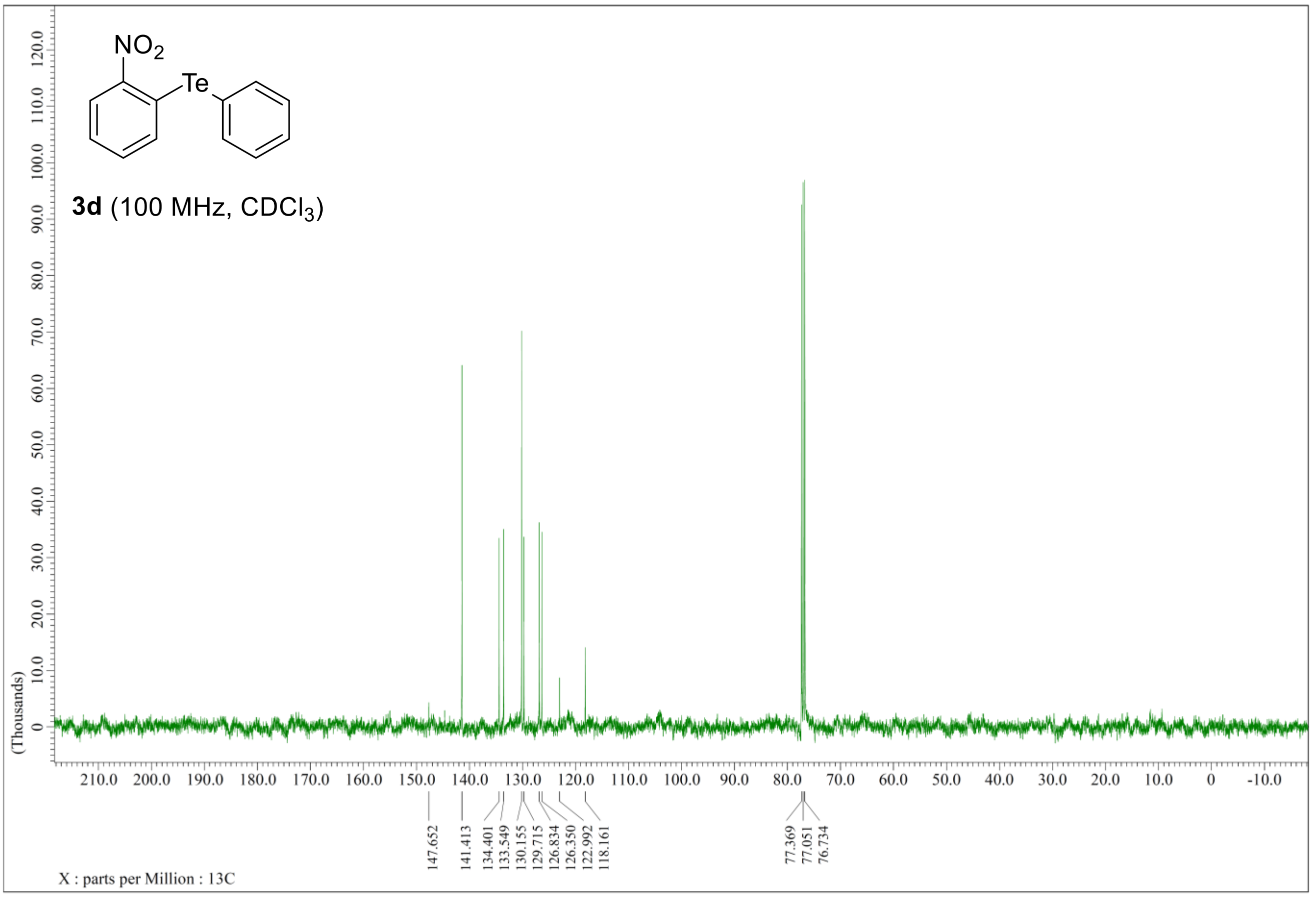
NO2
Te
3d (100 MHz, CDCl3)
147.652
141.413
134.401
133.549
130.155
129.715
126.834
126.350
122.992
118.161
77.369
77.051
76.734
(Thousands)
X : parts per Million : 13C

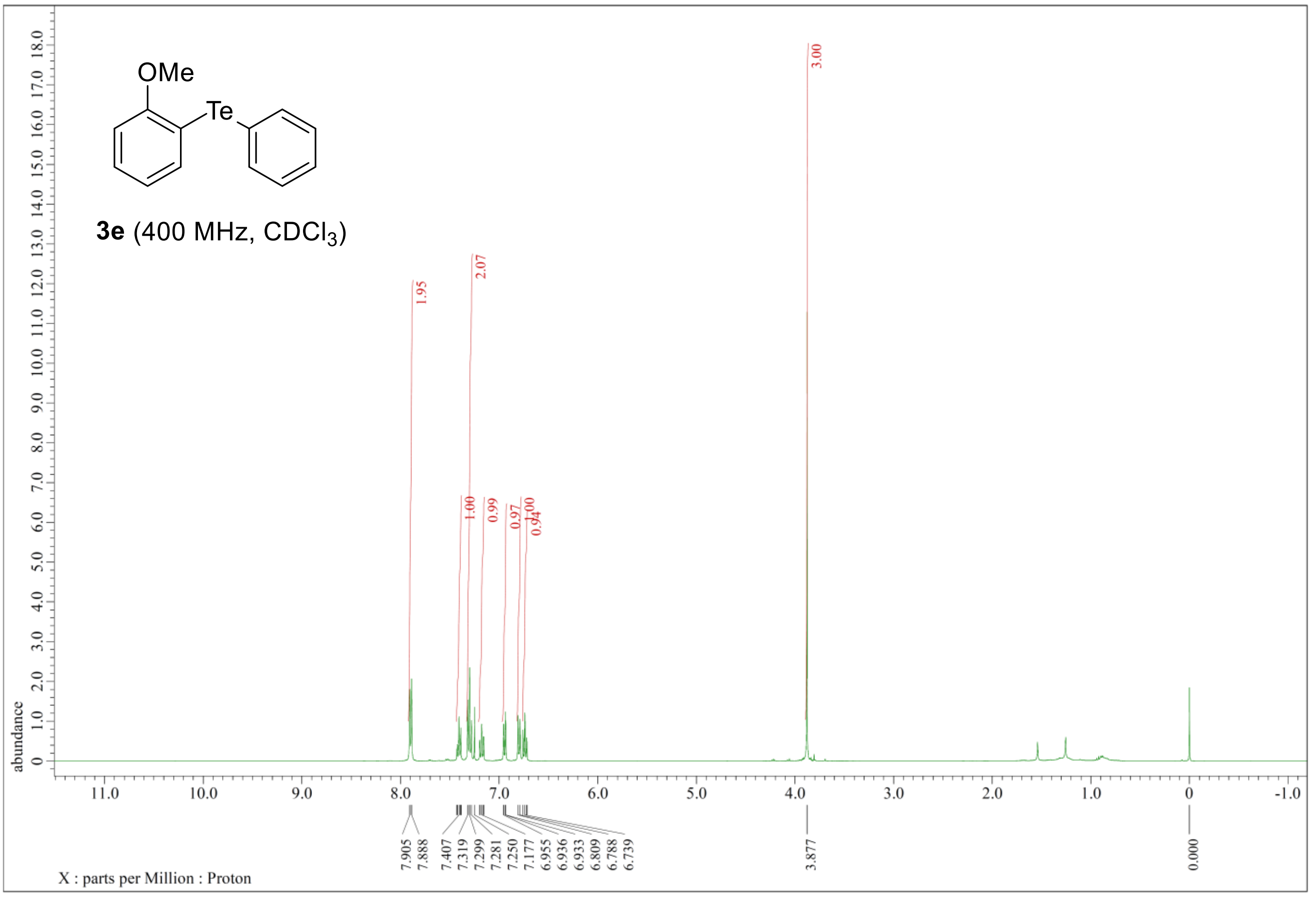
OMe
Te
3e (400 MHz, CDCl3)
abundance
X : parts per Million : Proton
7.905
7.888
7.407
7.319
7.299
7.281
7.250
7.177
6.955
6.936
6.933
6.809
6.788
6.739
3.877
0.000
1.95
2.07
1.00
0.99
0.97
1.00
0.94
3.00

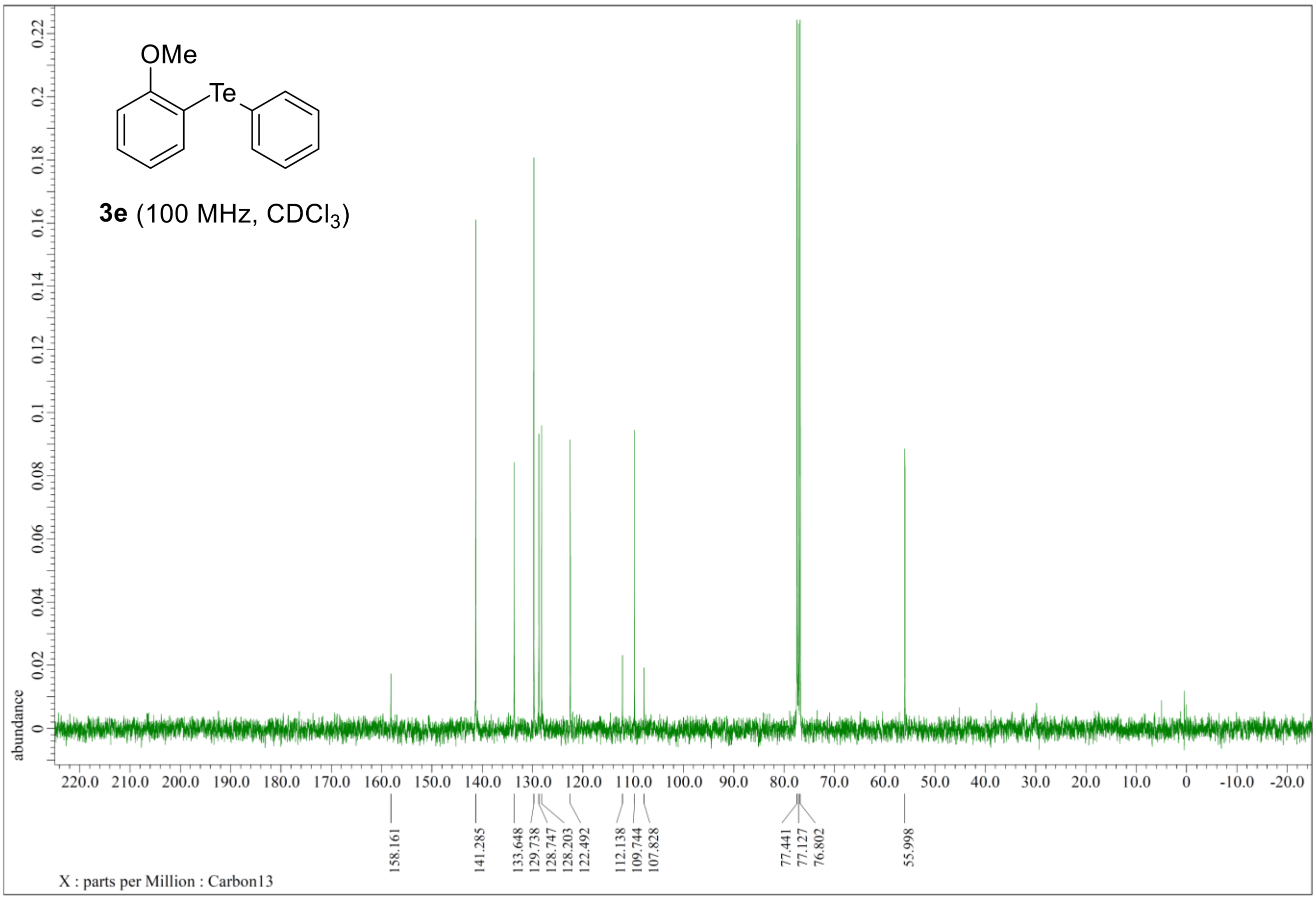
OMe
Te
3e (100 MHz, CDCl3)
abundance
X : parts per Million : Carbon13
158.161
141.285
133.648
129.738
128.747
128.203
122.492
112.138
109.744
107.828
77.441
77.127
76.802
55.998

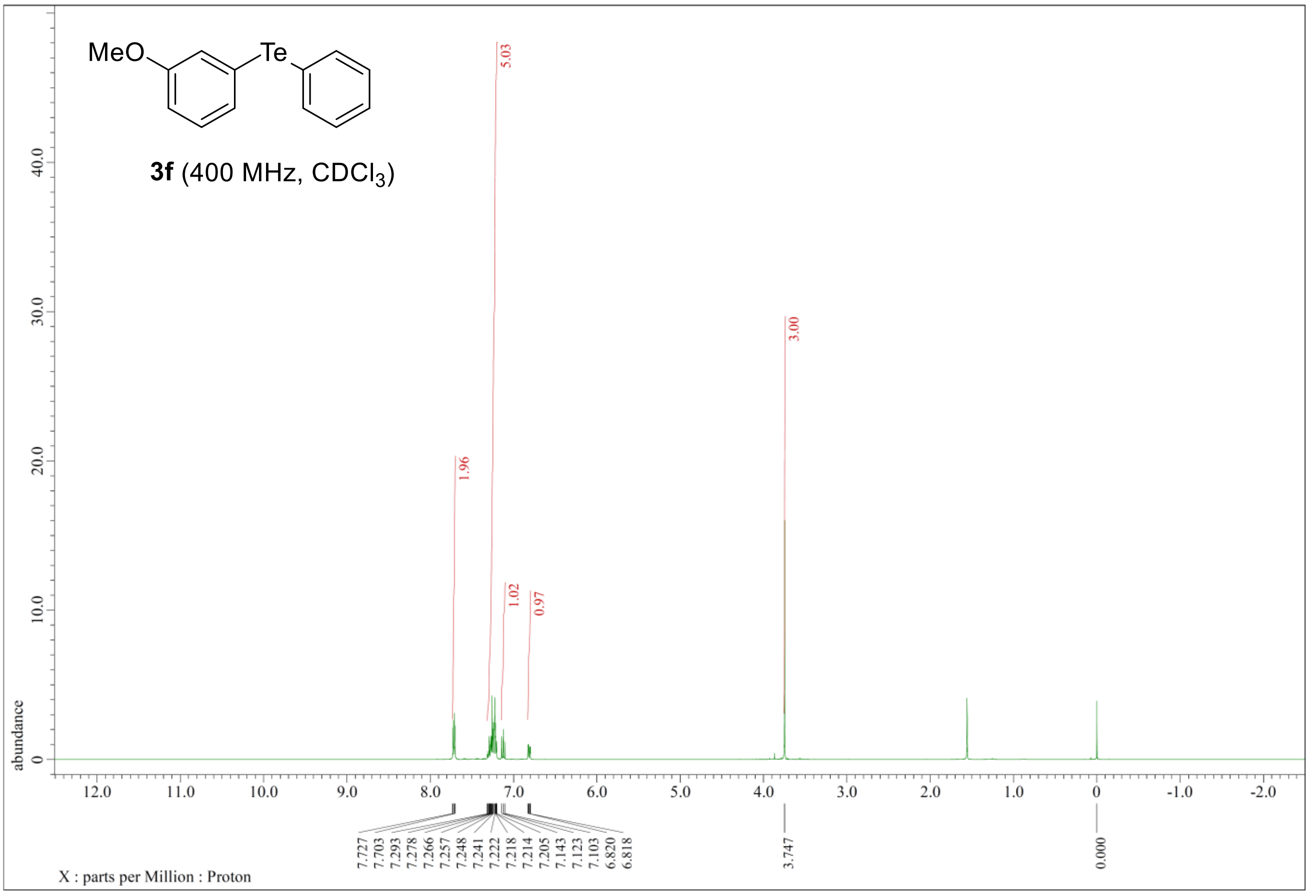
MeO
Te
3f (400 MHz, CDCl3)
abundance
X : parts per Million : Proton
3.747
0.000
1.96
5.03
1.02
0.97
3.00

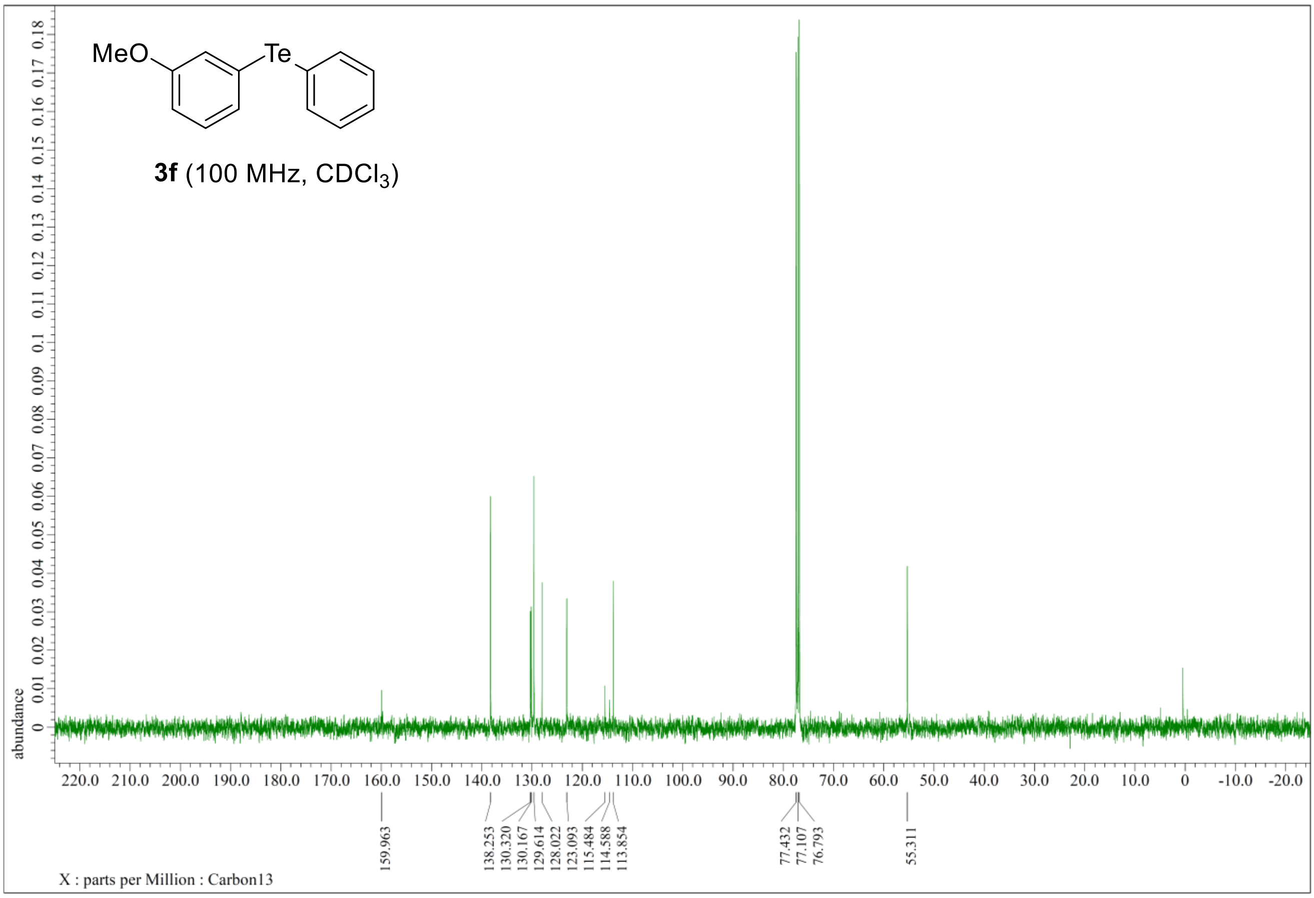
MeO
Te
3f (100 MHz, CDCl3)
abundance
X : parts per Million : Carbon13
159.963
138.253
130.320
130.167
129.614
128.022
123.093
115.484
114.588
113.854
77.432
77.107
76.793
55.311

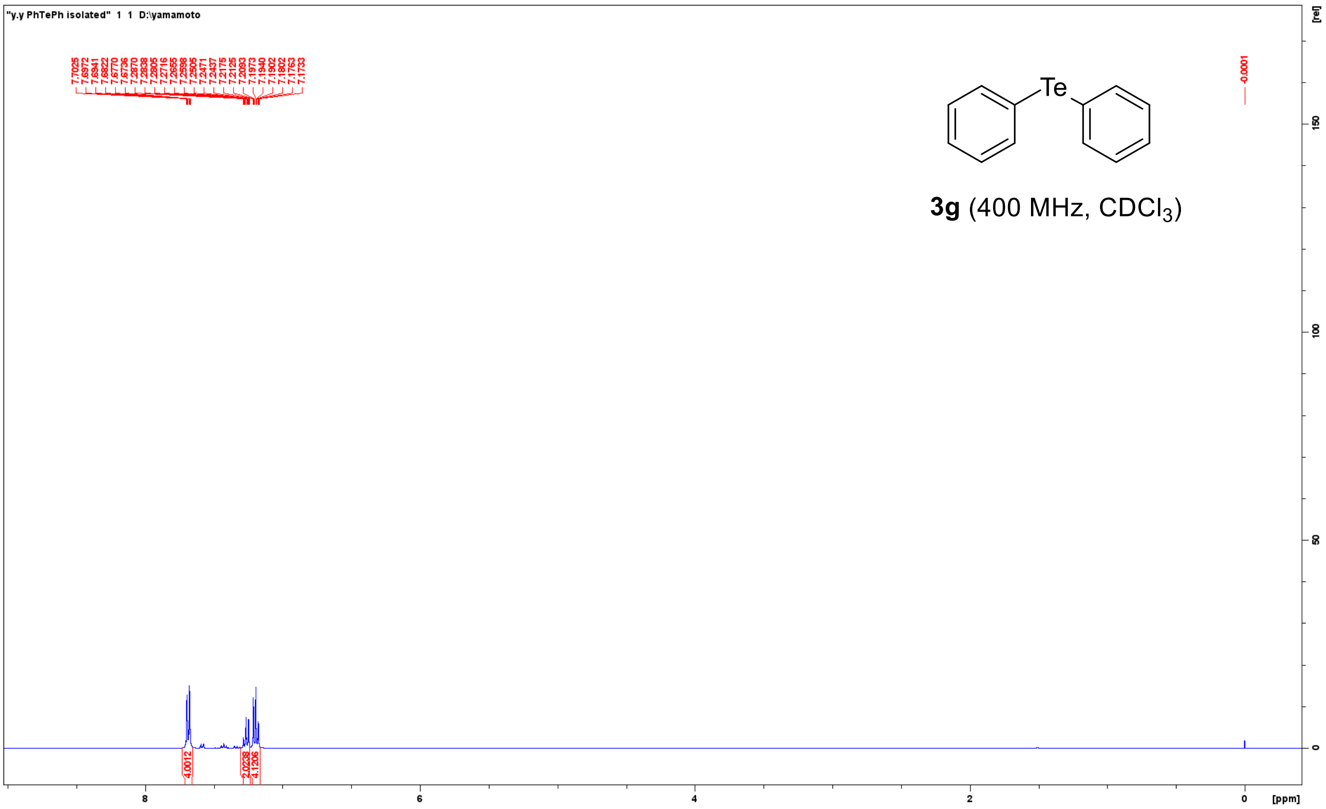
"y.y PhTePh isolated" 1 1 D:\yamamoto
Te
3g (400 MHz, $CDCl_3$)
[ppm]
[rel]

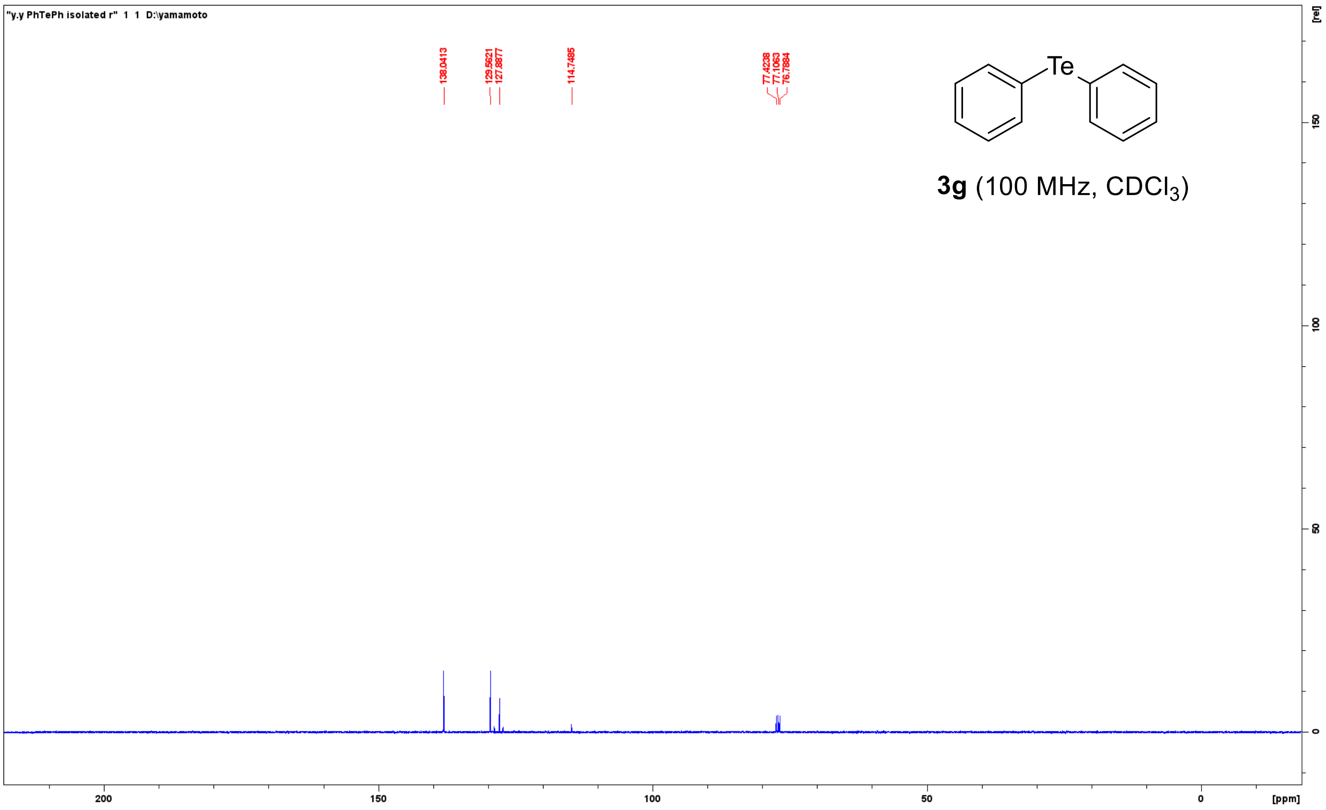
"y.y PhTePh isolated r" 1 1 D:\yamamoto
138.0413
129.5621
127.8877
114.7485
Te
3g (100 MHz, $CDCl_3$)
[ppm]
[rel]

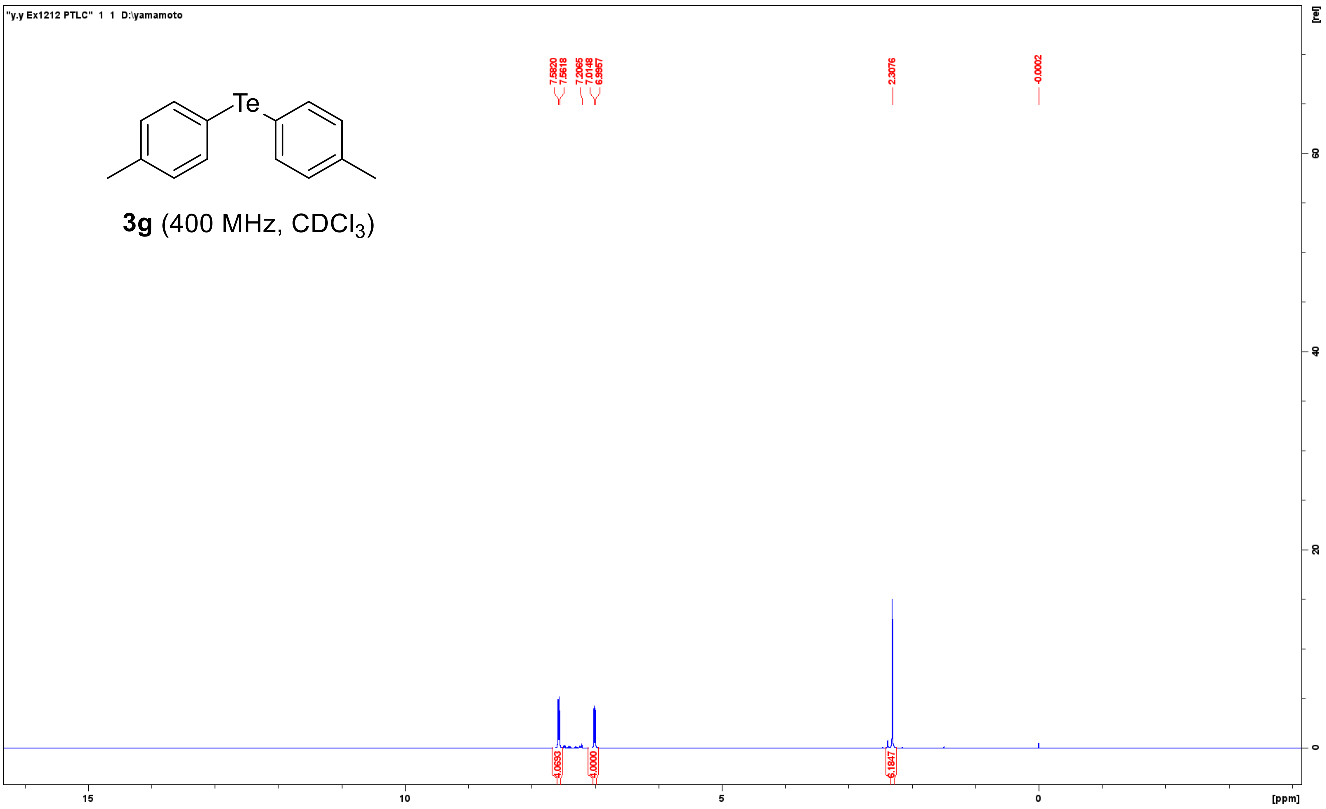
"y.y Ex1212 PTLC" 1 1 D:\yamamoto
Te
3g (400 MHz, CDCl3)
[rel]
[ppm]

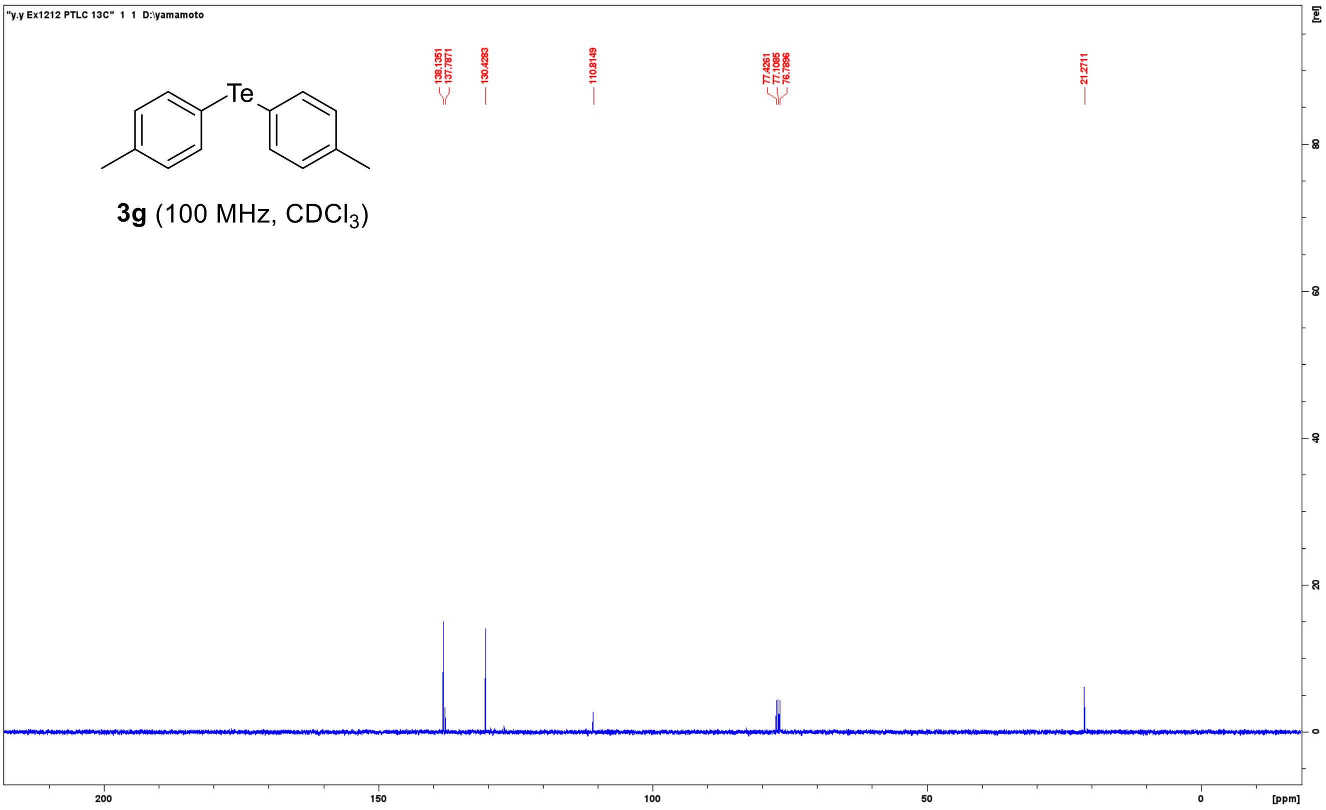
"y.y Ex1212 PTLC 13C" 1 1 D:\yamamoto
Te
3g (100 MHz, CDCl3)
[rel]
[ppm]

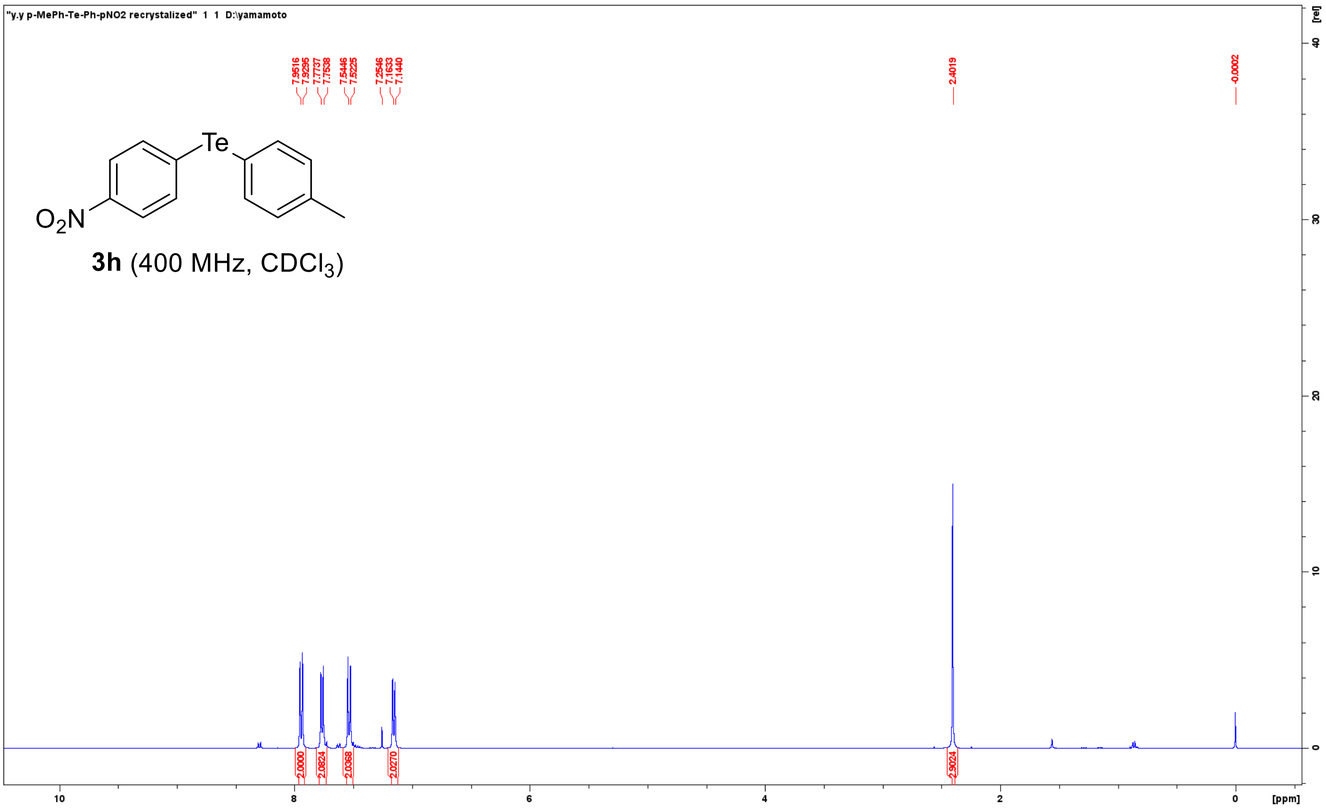
"y.y p-MePh-Te-Ph-pNO2 recrystalized" 1 1 D:\yamamoto
Te
O2N
3h (400 MHz, CDCl3)
[ppm]
[rel]

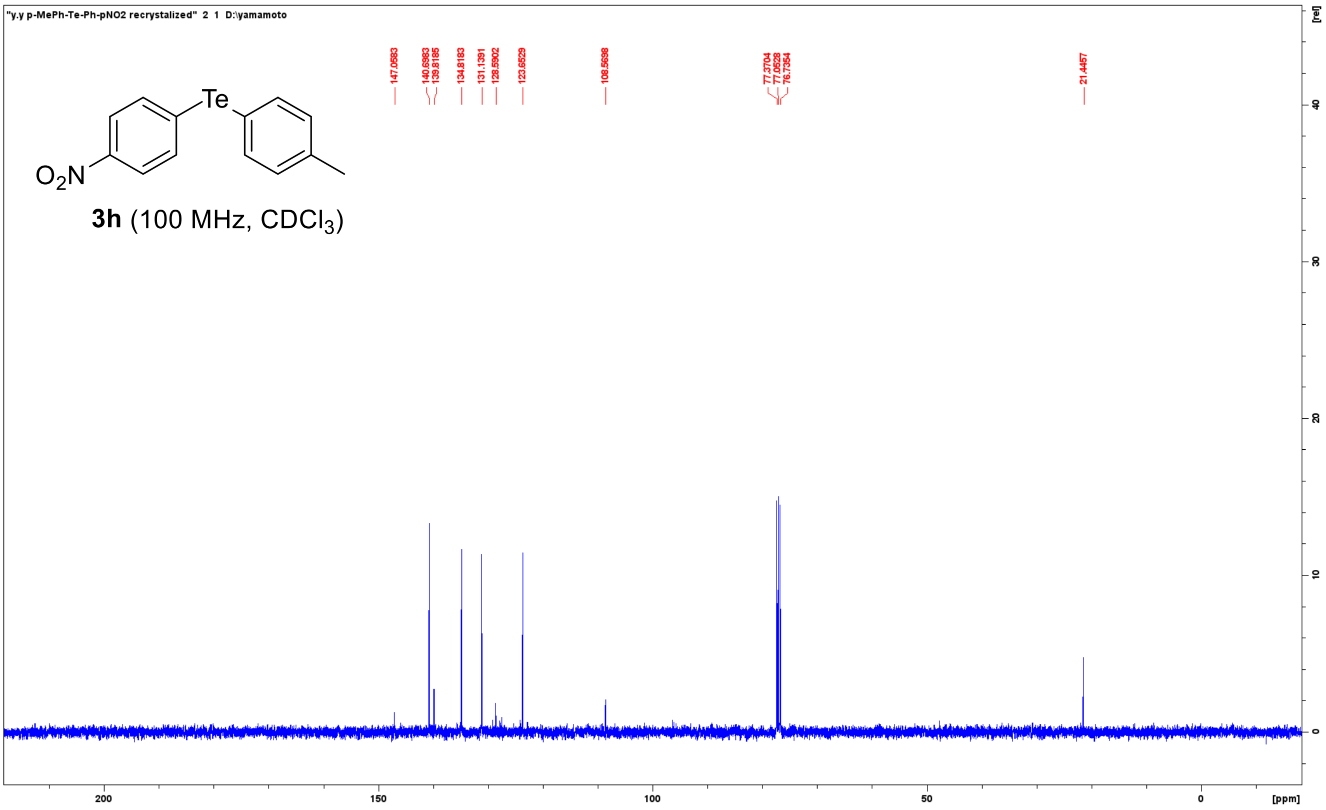
"y.y p-MePh-Te-Ph-pNO2 recrystalized" 2 1 D:\yamamoto
Te
O2N
3h (100 MHz, CDCl3)
[ppm]
[rel]

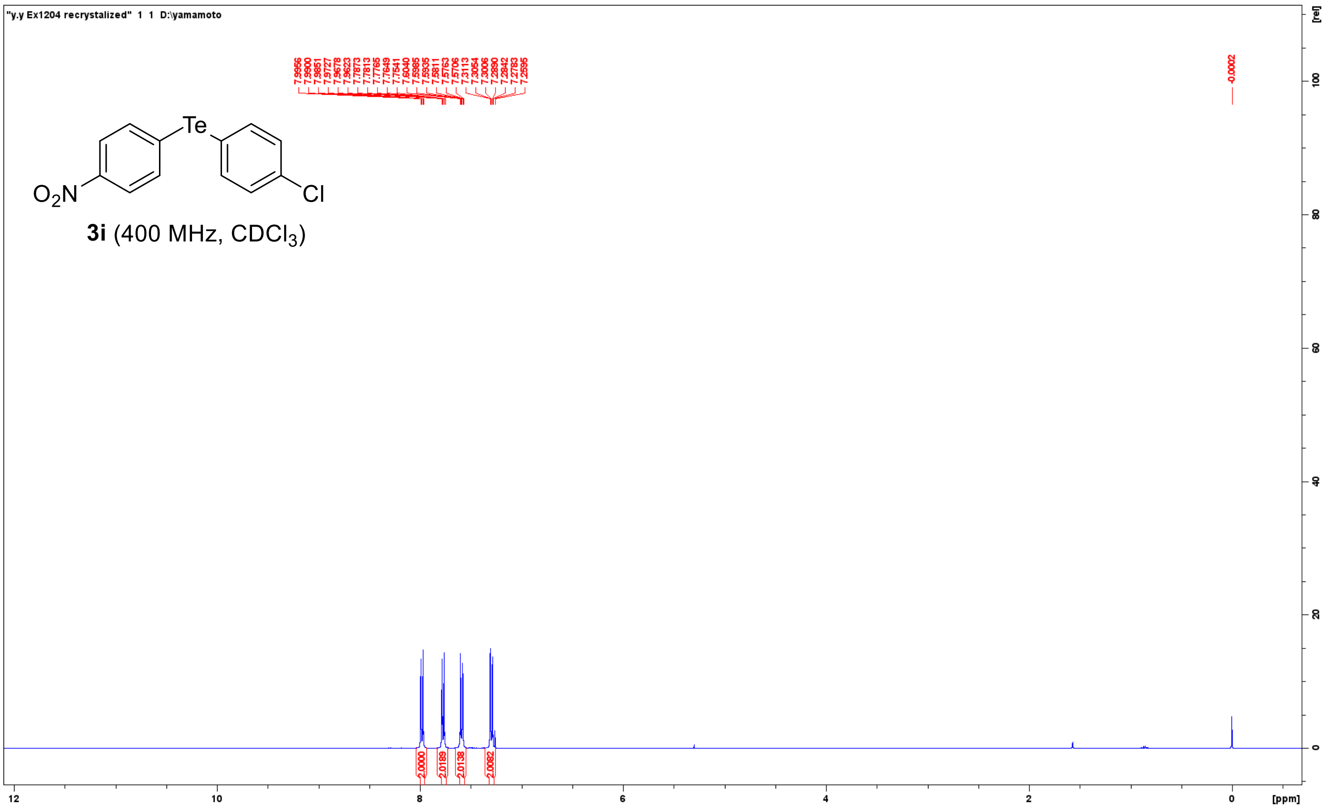
"y.y Ex1204 recrystalized" 1 1 D:\yamamoto
Te
O_2N
Cl
3i (400 MHz, $CDCl_3$)
-0.0002
2.0000
2.0189
2.0138
2.0062
[ppm]
[rel]

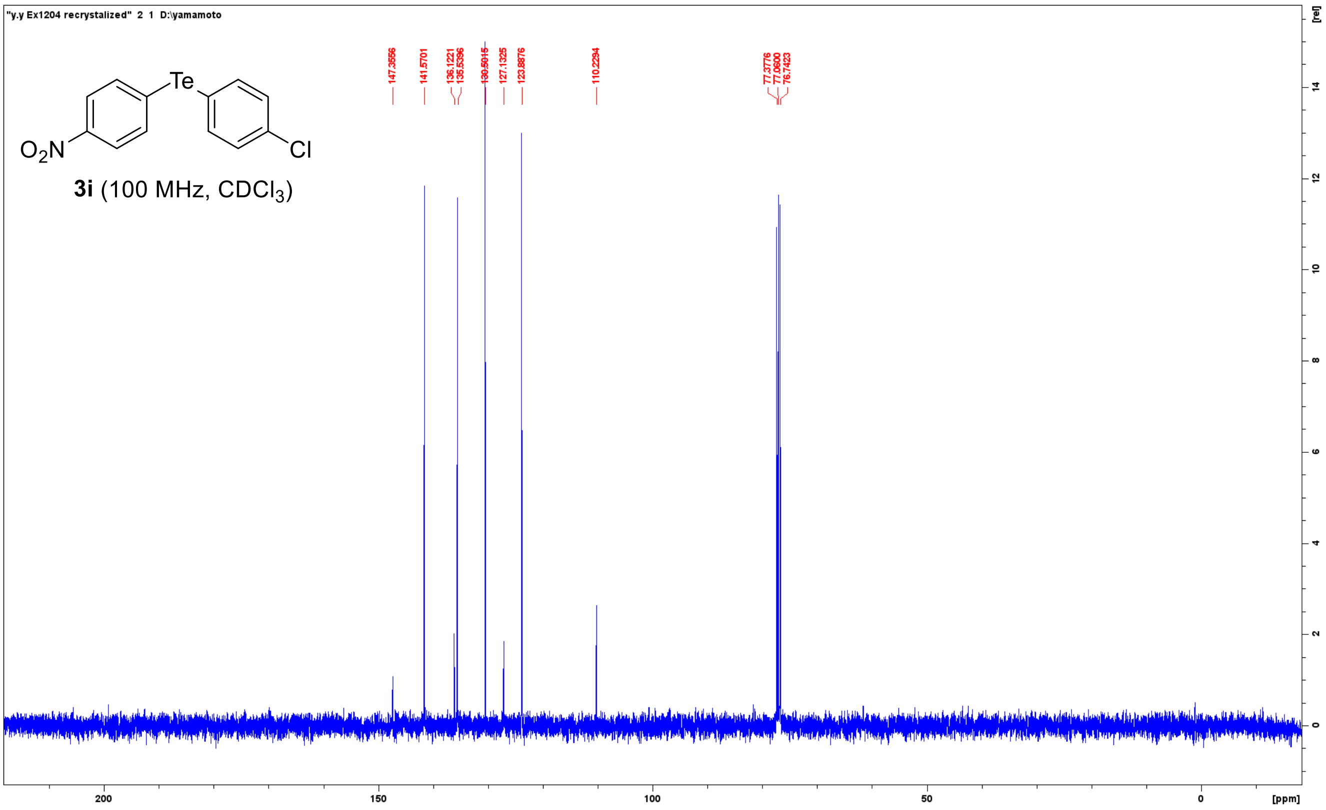
"y.y Ex1204 recrystalized" 2 1 D:\yamamoto
Te
O_2N
Cl
3i (100 MHz, $CDCl_3$)
147.3556
141.5701
136.1221
135.5356
130.5015
127.1325
123.8876
110.2294
77.3776
77.0600
76.7423
[ppm]
[rel]
